# Transactions of the Medical Society of the County of Kings

**Published:** 1865-04

**Authors:** A. N. Bell


BUFFALO

Medical and Surgical Journal.

VOL. IV. APRIL, 1865. No. 9

ART. I.—*Transactions of the Medical Society of the County of Kings.*

REGULAR MEETING, JANUARY, 1864.

*Disinfection—Paper by* DR. A. N. BELL.

The word *infection* is frequently used to indicate the vitiation of the atmosphere from any cause whatever. Hence the air is said to be infected when it contains an excess of carbonic acid or ammonia, or when its natural components do not exist in their usual proportion.

Such air is unquestionably bad, and is often the cause of ill health. But the diseases arising from these conditions have no special characteristics. A multitude of persons subjected to malaria of this kind will be variously affected, usually according to individual predisposition. But when a special disease, with characteristic symptoms, occurs as the result of malaria, or, above all, when of a multitude of persons exposed to malaria, a large number being taken sick in consequence of the exposure, are similarly affected, the poison in all such instances is putrefying organic matter. Hence I deduce the definition of infection to be a poisonous emanation of organic matter in a state of putrefaction, *which is incapable of reproduction in the human system.*

From this stand point I submit the following reflections: The existence of organic matter in the atmosphere is universal. It is everywhere the product of combustion and decay, and is given off by all animals in respiration. The processes of life, death and decomposition are accommodated to the whole of nature's domain. Latitude, elevation, nature of the soil, degree of cultivation, relative position in regard to mountains, forests, rivers, etc., and general aspect of the neighborhood, all modify the conditions of the atmosphere and man's liability to pervading influences. It is, therefore, just as natural that stagnation, dampness, darkness and high temperature should cause disease and death, as that a free circulation of pure air, light, dryness and moderate warmth should promote health and long life. And it is, also, just as natural that there should exist conditions favorable to death and putrefaction as that there should exist conditions favorable to vitality and health. The qualities of all natural phenomena have certain operations, each peculiar to itself, yet all in harmony with every other. And if we apply our knowledge of the laws of organization in tracing the causes of disease, we shall be no less able to escape from those which are amenable to destructive agents than we are from those which result from ignorance and misconduct. The smoldering alluvium of a tropical delta, bedarkened by a thick-leaved vegetation, and immersed in an almost perpetual fog, is, of all places, most prolific of infection. The putrefying mass is also a hot-bed for the production of innumerable species of short-lived fungi, and the myriad spores of these terminating their brief existence, commingle with the putrid emanations. The varying conditions of climate and season render these emanations insignificant at one time but deadly at another; and in this latter case persons are not only liable to immediate danger from respiration, but their clothing, the material of commerce, the bulkheads of vessels, furniture and cargo, are all subject to the pervading infection. The articles of commerce thus infected become fomites or retainers of infection, liable not only to communicate disease to persons in their proximity, but to become the leaven, as it were, of new places possessed of the fitting conditions of climate and domicil.

The virus of contagion, too, is unquestionably organic in its nature, and active only when in a state of putrefaction.

In its formal existence the virus of contagion cannot be distinguished from the virus of infection, but in its origin it has this peculiar difference, namely: it is reproduced by its kind in the human system, and communicable by one person to another, or by persons to things, which thereby become contagious fomites, or new sources of contagion. On account of this similarity in the manner of communication under certain circumstances by fomites, authors are wont to regard contagious diseases as being also infectious. Hence has arisen the frequent use of the words contagion and infection synonymously. Although not strictly germain to the purpose of this paper, the subject is nevertheless sufficiently pertinent to admit of the suggestion, that inasmuch as the virus of contagion does not lose its special character of reproduction in its progress through the human system by being also capable of lodgment in surrounding things, it should not on this account be confounded with other poisons that are devoid of this property. *The virus of contagion*, small pox, for example, whether contracted from fomites or a person affected with the disease, *is capable of reproduction in the system*, and liable to be communicated to other persons or to other things, and so on. But *the virus of infection* proper, that which arises from the processes of decomposition under certain climatic conditions and gives origin to such epidemics as yellow fever or cholera, *is wholly incapable of reproduction in the human system, and cannot, therefore, be transmitted by one person to another, or from the person to any substance.* The fomites of contagion and the fomites of infection are as much the representatives of the two great families of epidemic diseases, contagious and infectious, as are these diseases respectively themselves. And the poisons, although alike in being organic in their nature, are, nevertheless, as characteristically different in their origin, symptoms and progress, as are the different species of any other genus in the natural history of disease. To disarm fomites, to destroy the virus of contagion or of infection, to render harmless poisonous emanations in the atmosphere of a place, is to disinfect.

Innumerable substances have, from time to time been brought before the profession as DISINFECTANTS, but with the exception of chlorine and its compounds, they have most of them proved to be deodorants only, and do not, therefore, in this connection call for much consideration. Some of those which have attracted most attention, however, may be named:—Ledoyen's liquid, which consists of a solution of nitrate of lead; and Larmaudé's antimephitec, which, according to an examination made by M. M. Tardieu and Cozalis, appears to be made of a solution of sulphate of zinc, with a little sulphate of copper, in order to make it patentable. Both of these compounds were extensively used in Paris a few years ago to disinfect the sewers, but it ultimately turned out that their properties were deodorant only, and they are consequently now only rarely used for any other purpose. A new compound has been recently brought to notice under the name of "The Ridgewood Disinfecting Powder. A sample of this powder was presented to the N. Y. Academy of Medicine in July last, and referred to the Section on Public Health and Legal Medicine. A report was in due time submitted, from which we learn that the composition of the powder, as given by Mr. Napier, the chemist and president of the company manufacturing it, is as follows:

| | | |
|---|---|---|
| Carbonic acid, | 5 to 8 | per cent. |
| Sesquichloride of iron, | 2 | " |
| Lime from magnesian limestone, | 5 | " |
| Silicate of alumina (in the form of Fuller's earth) | 75 to 80 | " |
| Prepared charcoal, or ground pumice-stone, | 10 to 12 | " |
| Sulphate of potash or soda, | a trace. | |

Several experiments demonstrate this compound to be a powerful deodorant and antiseptic, but evidence is yet wanting of its disinfecting properties.

It is a common impression that because of the natural tendency of gases to rapidly permeate each other and become equally diffused, that therefore simple exposure to the atmosphere will in most cases necessarily overcome infection. But this is true only to a very limited extent. If pure air were fully and constantly accessible it would doubtless altogether prevent noxious emanations, partly by its dispersion of matter and partly by its chemical properties; tending constantly to dilute, disperse, and decompose all pernicious emanations from whatever source. But it was surely never intended by the Creator that an important natural phenomenon—the transition of matter—should either cease or be materially modified for the special benefit of one particular race of his creatures. On the contrary, it is manifest that there are many places both natural and artificial to which a sufficient supply of pure air for disinfection is inaccessible. The winds from the direction and in the immediate vicinity of such places are in some degree like the simoon of Africa and the sirocco of Italy, they are loaded with dangerous emanations from the localities over which they have passed. And as a general rule it is unsafe to be within two miles to the leeward of vessels or places known to be infected.

If infection were indeed a gas, simple exposure to the atmosphere, or, at any rate, to such gases as would then be known to effectually decompose it, would be infallible disinfectants. But the putrefying particles of organic matter, though light, are nevertheless heavier than atmospheric air, and their tendency is in consequence to occupy the lower strata. Hence cellars and the holds of vessels, from the very nature of their structure, cannot be so freely exposed to the atmosphere as to disinfect them, except at very long periods of time. The effect may be speedily manifest or an indefinite length of time may elapse, according to the conditions of the atmosphere and the degree of cleanliness, and the danger still exist. Under these circumstances and climatic conditions favorable to the spread of infection, it is manifestly absurd to undertake to limit the period of time by days when a room known to have been infected may be safely occupied, or an infected ship or cargo may be admitted to pratique.

Chlorine, nitrous, sulphurous and manganic acids, variously compounded with salts; and, recently, bromine, and numerous other gaseous substances which it is unnecessary to mention, are it is well known, extensively used as disinfectants. Such substances doubtless exercise a salutary influence over malaria depending upon the presence of deleterious gases, merely, which are by these means decomposed. But the influence which such gases and vapors exercise over the presence of putrefying organic matter, is probably only that of precipitation, hence, as experience shows, they are efficient only for very brief periods of time. Against inorganic matter, precipitation, even by watery vapor alone is, under certain circumstances, a commendable prophylactic. Mr. Leopold Brandeis, a scientific man of this city, who is engaged in the manufacture of *lead-pipe*, informs me that his workmen never have lead colic; and he attributes their exemption from that disease to the circumstance that special provision is made for the continued escape of a small amount of steam from the machinery, for the purpose of precipitating the minute particles of lead which would otherwise float in the atmosphere of the factory, and thus be subject to inhalation. But against putrefying organic matter, vapor, with the warmth necessary to generate it, is worse than useless.

Of all the gaseous and vaporous substances hitherto used for disinfecting purposes, *chlorine* is unquestionably the most potent and penetrating. The utility of it, therefore, may be taken as a fair example of the highest degree of benefit to be expected from the use of this class of agents.

Chlorine by its superior affinity for hydrogen seizes upon this element of gaseous compounds, and thus decomposes while it deodorizes them. But unless highly concentrated and kept up for a long period of time, there is abundant evidence demonstrating that chlorine has no such power over non-gaseous matter, such as the true virus of infection or contagion. The following examples which fell under my observation during the summer of 1862, are appropriate illustrations:

The steamship *Khersonese* arrived at New York, August 17, four days from Bermuda, a healthy port. She had been in quarantine at Bermuda twenty-four days, and had lost in all since leaving Nassau, an infected port—her last port of departure—some six weeks before her arrival here, ten persons with yellow fever. On arrival she had no sickness on board, but, having had it, she was "fumigated" with chlorine, and allowed anchorage at upper quarantine. Three days afterwards she had a case of yellow fever. Fifteen days afterwards, and after she had discharged ballast, been again "fumigated," and taken in cargo, she had two other cases. She shortly afterwards departed. The steamer *Dispatch* arrived August 29, four days from Nassau. She had lost five men by yellow fever, and on arrival had four cases. She was repeatedly "fumigated," the hatches kept off and part of her cargo taken out at lower quarantine. No new case having occurred after two weeks' detention, she was permitted to go to upper quarantine, discharge balance of cargo, and after thorough *chlorination*, permitted to re-load. September 29th, just one month from the time of her arrival, she had a new and very malignant case that died with black vomit on the third day. Chlorine, besides, is often inapplicable on account of its destructive properties to the material of fomites.

*Water*, under some circumstances, is a valuable disinfectant. Although moisture associated with other conditions is rapidly promotive of putrefaction, and the propagation of fungi, tending to perpetuate the mischief; yet total submersion involves a different train of circumstances of a far less noxious character. Organic matter by maceration in water is oxydized, and among other products nitric acid is generated, which is antiseptic. Everybody knows that if a marsh is continually submerged it is far less dangerous than when subject to ebb and flow; especially is this the case if the water is cold. If the water is warm, organic matters in a state of decay are liable to be borne off with the vapors, and so become injurious. *Soil,* too, is a certain but slow disinfectant. The interment of fomites, like maceration in water, can be practiced only to a limited degree. *Cold,* when of sufficient intensity is a powerful disinfectant and antiseptic. The iced-up animals of the frigid zone are an example. And the recurrent seasons of winter, it is well known, effectually arrest epidemic non-contagious diseases in temperate latitudes.

Infection subjected to a freezing temperature, even for a short period of time, is effectually destroyed; but the difficulty consists in the application of the necessary degree at the proper time. Infection pervades the closest textures, every seam and crevice. How is it possible in the midst of a warm external atmosphere, and the waters of the gulf stream to apply a freezing temperature to the whole interior of a ship and cargo? Of many examples known to the writer, of the futility of artificial cold to infected vessels, the following one will suffice: April 15th, 1858, the U. S. Steamer *Susquehanna* arrived at New York infected with yellow fever. After about sixty days' detention, and after the weather had become very hot, she was ordered by the Health Officer to be broken out for the purpose of freezing, by means of ice put on board. The experiment cost the government over $20,000 and many valuable lives. She continued to have cases of yellow fever on board and was not admitted to pratique until frost in November. It has been the common practice of the navy department, in peaceful times, to order vessels that have had yellow fever on board, to lie in some northern port during the next succeeding winter. It is scarcely necessary to add that this is impracticable in time of war, and at all times to the merchant. Infection, whenever and wherever it is found to exist, should be avoided or destroyed; and the means used for this latter purpose, should be, if possible, speedy, certain and practicable at all seasons and places. And to this end, there is reason to believe that of all the means hitherto used for the purpose of disinfection, the most efficacious is *heat*.

Epidemics, it is well known, rarely prevail during an average temperature above 85° Fahrenheit. In Egypt, the plague is effectually arrested by the heat of midsummer. Putrefaction ceases, and mummies are preserved in the burning sands for an indefinite period. Dryness, doubtless has much to do with this, while the circumstance serves to indicate the utility of "a fire in the camp," which, in tropical marshes is proverbial for its sanitary influence.

Impressed with the utility of heat as a disinfectant, Dr. William Henry, F. R. S., of Manchester, as long ago as the year 1824, instituted a series of experiments to test its effects upon the "contagious element" of small pox. Dr. Henry's first series of experiments satisfactorily established the fact "that the infectious matter of cow-pox is rendered inert by a temperature of 140° Fahrenheit," from whence he inferred that more active contagions are probably destructible at temperatures not exceeding 212° Fahrenheit. His next series of experiments were upon the personal fomites of typhus and scarlet fever. Three flannel shirts, taken on three successive days, from a strongly marked case of typhus fever, were subjected to 204° Fahrenheit for an hour and three-quarters. These personal fomites being before the application of heat as thoroughly charged with the contagious principle as any garment could be, were tested as follows: "One was placed directly under and within twelve inches of the nostrils of a person engaged in writing and who was excessively fatigued from previous exercise and had observed an unbroken fast for eight hours. This test of exposure was continued for two hours. The second shirt was put on and worn next to the body of a person for two hours. And the third, with the view of giving activity to any contagious matter which might possibly have escaped decomposition, was put into an air-tight canister for twenty-six days. It was then taken out and placed within twelve inches of the face of a person for four hours, a gentle current being contrived to blow upon him from the flannel during the whole time. In none of these instances was the fever communicated, and no injurious effects were experienced." Dr. Henry next performed a precisely similar series of experiments with the fomites of scarlet fever which proved to his satisfaction "that by exposure to a temperature not below 200° Fahrenheit, during at least one hour, the contagious matter of scarlatina is either dissipated or destroyed." And he remarks "the circumstances under which the experiments were conducted, render it, I think, demonstrable that the disinfecting agency belongs to heat alone; for the receptacle in which the infected waistcoats were placed, having in every instance been closed, change of air could have had no share in the effect. The phenomena then are reduced to their simplest form and the results put us in possession of a disinfecting agent the most searching that nature affords—one that penetrates into the inmost recesses of matter in all its various states." Having satisfied himself in this direction, Dr. Henry next undertook to ascertain what elevation of temperature "cotton and other substances likely to harbor contagion of the plague or typhus, would sustain without injury, the heat being applied to both the raw staples and to their various fabrics. A quantity of raw cotton, subjected to a dry temperature of 190° Fahrenheit, which was steadily kept up in the inner compartment of a double vessel heated by steam during two hours, became fuzzy on account of the loss of its natural moisture, and for the same cause the strength of the yarn was for the time impaired; but after being left for two or three days in a room without fire, a great change had taken place in its appearance, and it was found on trial that the cotton was as capable of being spun into perfect yarn as that originally employed. On accurate trial of the twist which had been spun from it, a hank supported an equal weight with a hank of the same fineness that had been spun from fresh cotton from the bag. This fact established by repeated experiments, proves that with the recovery of its hygrometrical moisture, cotton which had been heated regains its tenacity and becomes as fit as ever for being applied to manufacturing purposes." A quantity of cotton yarn was tested in like manner with like result. "Articles of cotton, silk and wool, after being manufactured, both separately and in a mixed state, into piece goods for clothing, were submitted to the same treatment. And some of these were of the most fugitive colors and delicate textures, yet after being exposed three hours to a dry heat of 180°Fahrenheit, and then left a few hours in a cool room, they were pronounced perfectly uninjured in every respect. Furs and feathers similarly heated were also uninjured. In subsequent experiment the temperatures were raised forty or fifty degrees higher without injury to the fabrics.* Crude sugar will stand a dry temperature of 200° Fahrenheit for an indefinite length of time without injury.

Dr. Von Busch, of Berlin, having the benefit of Dr. Henry's experiments, in February and March, 1851, after having ineffectually made all the usual appliances, thorough cleansing, aeration, fumigation, etc., for the purpose of disinfecting the Berlin Lying-in Hospital of puerperal fever, determined to try the effect of dry heat. All the beds, wardrobes and hospital utensils being retained in the wards, common wood stoves were introduced and a steady temperature of about 150° Fahrenheit was kept up for two days. The wards were immediately re-occupied by the same class of patients, with the same individual liabilities as before, and the result was found to be triumphant. The infection was destroyed and the inmates were safe. A subsequent return of the disease on the following year, was destroyed in the same manner.†

A striking instance of the disinfecting power of heat to a badly infected ship, is referred to in vol. viii of the Royal Medico-Chirurgical Transactions, asbeing contained in the official report of Dr. William Ferguson, inspector-general and chief medical director for many years, in the Windward and Leeward Islands. The reference states that the transport ship Regalia, being badly infected with yellow fever while at English Harbor, underwent fumigations without the least effect in arresting future attacks or their fatality; and that it was not until after her arrival in Carlisle Bay, where she was completely cleared, and, with the hatches closed and her whole hold exposed to the concentrated heat of many stoves, that fever ceased.

Heat is no less efficacious in the form of steam or hot water. In a paper on this subject by Dr. Elisha Harris,‡ before the Fourth National Quarantine and Sanitary Convention, he states that:—

* Philosophical Magazine, 1831–32.

† Neue Zeitschrift, Fur Gebeertskunde, Berlin, 1852. Bull. de Therapeut, 1853.

‡ Utility and Application of Heat as a Disinfectant, 1860.

"During a period of nearly fifty years, the washing and drying of the contaminated clothing from hospital patients and infected vessels had been performed in the ordinary way, without the use of steam. The diffusion of fatal fevers from these fomites of infection was notorious during that protracted period. Immediately after the introduction of steam tubs for boiling and a steam-heated chamber for drying the clothing, and obviously as a result of those improvements, the occurrence of infectious or quarantine diseases among the washer-women of that establishment ceased, or at least they occurred but very rarely, and then from sources to which the steam heat had not been applied.

"Early in the summer of 1856, when large quantities of dunnage were ordered to the wash-house from vessels infected with yellow fever, I ascertained that the two washer-women who were attacked with that malady had been handling and washing various articles of clothing previous to steaming or boiling them. Though those unfortunate washers might have contracted the fever elsewhere than in the wash-room, it was deemed expedient to use greater precautions against infection, and accordingly directions were given that all clothing, both from ships and hospitals, should be steamed in the closed tubs previous to being distributed to the washers. Infected dunnage and clothing continued to be received in large quantities for several months subsequent to that order, but no more cases of yellow fever occurred among the washers."

Again, in the summer of 1859, a floating hospital was placed under my superintendence for the reception and care of all cases of yellow fever and other pestilential diseases arriving at the port of New York. The practice of burning all dunnage, bedding and other clothing from infected vessels, having obtained favor with the authorities who witnessed the same expensive and unsatisfactory process applied to the entire quarantine establishment, it had been advised that a like summary method of purification be continued in connection with the hospital ship; the famous iron scow for the burning of infected ships' clothing, bedding and dunnage, being still in existence. Accordingly, no apparatus or provision of any kind had been placed on board for the cleansing or for the reception and proper care of infected ships' clothing, nor even for the washing and preservation of the clothing of the patients and their bedding. The hospital ship had already been placed at the yellow fever anchorage, twenty miles from the city, and was awaiting the arrival of the sick with fever. Under these circumstances, a wash-room was, under my direction, hastily extemporized, furnished with a copper steam-generator and capacious steam-vats, steam-washtubs, etc. This apparatus was placed in onc of the galleries that had previously been constructed upon the outside of the vessel amidships, and to the after end of each of which, entrance was made by the gangway, outside, both from boats and the wards.

"Into the steam-vats was thrown every infected thing received from vessels as well as all hospital and patient's clothing, etc., that required cleansing. All articles from infected vessels were received directly into the steam chamber from boats without entering the ship itself, or in any manner exposing it or its inmates to the danger of infectious contamination; while in the wards of the hospital a like safe regulation was adopted, requiring every article as soon as soiled to be removed to the steam vats, and there all substances capable of being febrile fomites were instantaneously heated to the boiling point or even a higher temperature.

"It will be observed that these arrangements contemplated the preservation of both the clothing and the wards from becoming fomites or foci of infection. The prediction having been reiterated by many persons that the hospital ship would certainly become infected, and be in itself a focus of pestilence, we are happy now to record the fact that with twelve cases of yellow fever, and with twelve cases of other maladies far more liable to personal or fometic communication, there was not an hour of sickness among all the employees of the floating hospital during the six months it continued in service, though the washer-woman and two other employees had never suffered from yellow fever, and had no specific protection from any disease except small pox."

The experiences of the floating hospital since Dr. Harris' superintendence have been equally favorable.

It is conceded that of all fomites a foul ship is the most persistent and the most to be dreaded. During the summer of 1847, almost every vessel of the U. S. Naval Squadron, in the vicinity of Vera Cruz, became infected with yellow fever. Among the rest, the steamer *Vixen*, which vessel had had a good deal of river service, was very filthy and infested with vermin, and was so badly infected toward the latter part of the season that all hands were constrained to sleep on deck. Though yellow fever ceased to *prevail* during the season of the northers, the winter months, the crew of the *Vixen* continued to be in a sickly condition, with an occasional case of fever sufficiently typical to remind us that "Yellow Jack" had not departed. Before the return of hot weather, about the 1st of May, 1848, there being no immediate prospect of going north, it was determined to break out, as far as practicable, while on sea service and paint ship. Previous to undertaking this, however, the commander, the late James H. Ward, Esq., resolved upon a final effort for the extermination of the vermin by steam. Everything liable to injury was taken on deck, the hatches closed, and by means of a common leather hose connection steam was turned in below decks. This was kept up for two or three hours, so that every crevice was completely permeated. After this there was a thorough scraping, white-washing and painting. There was an immediate improvement in the health of the crew, and not another case of fever to the end of the cruise in midsummer. A few weeks subsequent to the steaming of the *Vixen* the gunboat *Mahones*, Commander W. D. Porter, having been on a surveying expedition up the Tuxpan river, returned to the anchorage at the mouth of that river, and telegraphed for the medical officer of the *Vixen* to visit the sick. There I found three cases of yellow fever, and within a few days four others occurred. The *Mahones* was a captured vessel from the Mexicans, had never been off the coast, and was filthy in the extreme. The salutary effects of the steaming on board the *Vixen*, both for vermin and fomites—no unusual associates, by the way—were so apparent, that the same process was forthwith advised and applied by means of the *Vixen's* engine and hose, to the *Mahonese*, and, as in the first instance, fever and vermin both ceased to exist; there was not another case. These vessels both continued on service in the vicinity of Vera Cruz until the following August, when they were sent to Norfolk, and were at once admitted to pratique. The *Mahonese* was there laid up until sold. The *Vixen*, after remaining three weeks without "breaking out," was, with a new crew, transferred to the coast survey in the Chesapeake Bay for the remainder of the summer. In neither of these vessels was there any return of the fever. About the same time that the *Vixen* and *Mahonese* arrived at Norfolk, the frigate *Cumberland* and the steamer *Scorpion* arrived at New York. The *Scorpion* was at once quarantined on account of recent cases of yellow fever on board; and the *Cumberland*, not having had any cases since the previous season, was, after "fumigation" and a few days' detention, permitted to go up to the Navy Yard to "break out." But scarcely had the work commenced before the yellow fever also broke out on board, and the vessel was, in consequence, sent down to quarantine and there kept until frost. The *Cumberland* and *Scorpion* were of the same squadron as the *Vixen* and *Mahones*, were more commodious, better ventilated and in every respect in better condition for health, *excepting that they had not been steamed.* Deeply impressed with the benefit of heat as applied in the cases of the *Vixen* and *Mahones*, I have frequently commended it; but until during my superintendency of the floating hospital, summer before last, I am not aware of its having been put in practice. Of all the infected vessels that arrived at this port during that season, the steamer *Delaware* was probably the worst; at any rate, the malignancy of the fever from that vessel was greater than that from any other. The *Delaware* had proceeded from the Tortugas in the early part of August with invalid soldiers on board, stopped at Key West, Fernandina, St. Augustine and Port Royal, where, in consequence of having yellow fever on board, she was put in quarantine twelve days, and then sent to New York. She arrived here September 21, having lost one man with the fever on the passage from Port Royal. On arrival, her commander, Capt. James S. Cannon and two of the crew, were sent to the floating hospital, and within five days afterwards seven of the invalid soldiers, all well-marked cases of yellow fever, and some of them so malignant as to have black vomit supervene within a few hours from the time of attack and to die within forty-eight hours. One died on board the *Delaware* within twenty-four hours, his case being so malignant that the boarding-officer deemed it useless to transfer him. In this state of affairs, at my urgent request, the remainder of the invalid soldiers (18,) were transferred to the (yellow fever) floating hospital, for safety.

They all escaped the disease; and I have not the least doubt that if all the soldiers had been removed on arrival, several lives might have been saved instead of lost by depending upon the effects of "fumigation." During the convalescence of Captain Cannon, I recommended to him the use of steam for the purpose of effectually disinfecting his vessel. I subsequently received the following letter:

"U. S. TRANSPORT 'DELAWARE,'
*New York, November* 30, 1862.

"DR. BELL:

"Dear Sir:—During my confinement in Quarantine Hospital with yellow fever, last summer, you suggested the idea of disinfecting my vessel by steam. In accordance with the suggestion, before my recovery the engineer steamed the lower cabin where nearly all the sick had been confined. After my recovery I more effectually steamed the vessel by closing her up below and driving the steam through her lower hold and bilges. This I did by attaching a hose to the boiler and leading it below through an aperture left for that purpose. Although we remained in quarantine three weeks after the first steaming, we had no sickness among a crew of twenty persons; and since that time the steamer *Delaware* has been in a perfectly healthy state. After refitting, the *Delaware* was sent to Port Royal with soldiers and encountered a heavy gale; of course everything was damp, but no sickness occurred on board and the troops remained perfectly healthy after landing. On my return, over a hundred invalid soldiers came north with me, but there was no sickness among them except that which they brought from the hospitals. The only injury resulting from the use of the steam was to the paint, which is stained; and, the first time charring the leather, and the second time melting the rubber hose. In using steam, hose which cannot be affected by heat ought to be provided especially for the purpose, by a copper coupling about ten feet long, attached to the cock where the steams comes directly from the boiler, and the heat is most intense. Much injury might otherwise result from the cracking of the hose, if leather, or melting it, if rubber, by the escape of steam.

"I am so well satisfied of the beneficial effects of steam on shipboard, that I would be sure of cleaning my vessel of that dread disease, the yellow fever, by its use in a very short time.

I am, very respectfully,

Your obedient servant,

JAMES S. CANNON,

*Master U. S. Transport Delaware.*"

From the foregoing premises the following conclusions are deducible:

1.—The various compounds, including chlorine and the salts which evolve it, hitherto used as disinfectants, have generally failed in their purpose; and probably because of the organic and non-gaseous nature of the virus which it is essential to destroy.

2.—That inasmuch as a temperature of 145° Fahrenheit, which coagulates albumen, if kept up for forty-eight hours effectually disinfects the worst fomites, we have in this fact alone strong evidence of the identity of virus with organic matter.

3.—That the necessary degree of heat for disinfection may be applied in some forms to almost every article of commerce or apparal liable to the virus of infection or contagion, without injury.

4.—That the examples furnished are an amply sufficient guide for the application of heat under the most variable circumstances.

NOTE.—Part of the material entering into the composition of this paper, was published in *Hunt's Merchants' Magazine* in October last, under the title of *Disinfection of Vessels.*

